# Management of STEMI in an Ectatic and Tortuous Right Coronary Artery Using a Flexible Guide Catheter Extension: A Case Report

**DOI:** 10.1155/cric/5644505

**Published:** 2026-04-18

**Authors:** Yasuyuki Toyama, Shinya Okabe, Tetsuya Hashimoto

**Affiliations:** ^1^ Department of Cardiovascular Medicine, Iseikai International General Hospital, Osaka, Japan

## Abstract

**Background and Aims:**

Coronary artery ectasia poses unique challenges during primary percutaneous coronary intervention for ST‐segment elevation myocardial infarction (STEMI), owing to thrombus burden and vascular tortuosity. This case demonstrates successful reperfusion using a flexible, hydrophilic‐coated guide catheter extension (GuidePlus, Nipro, Osaka, Japan).

**Methods:**

A 45‐year‐old man with inferior STEMI had complete occlusion of a severely ectatic (6.5 mm, 1.8× reference) and tortuous RCA with a ‘shepherd’s crook’ morphology. The conventional GuideLiner failed to cross. GuidePlus, a soft and hydrophilic extension, enabled thrombus aspiration and intracoronary urokinase (240,000 IU) delivery. A 4.0 × 28‐mm drug‐eluting stent (Ultimaster, Terumo) was deployed and postdilated (5.0‐mm NC balloon).

**Results:**

The GuidePlus enabled smooth navigation, direct aspiration and thrombolysis. Final angiography confirmed TIMI Grade 3 flow with minimal residual thrombus. The patient was discharged uneventfully and has remained event‐free with dual antiplatelet therapy (aspirin and prasugrel).

**Conclusion:**

GuidePlus offers excellent flexibility and crossability for managing STEMI in ectatic and tortuous arteries when conventional devices fail.

## 1. Introduction

Coronary artery ectasia (CAE) is characterised by aneurysmal dilation of the coronary arteries and is defined as a diameter at least 1.5 times larger than normal. It is observed in 0.3%–4.9% of coronary angiograms and predisposes patients to thrombus formation and subsequent acute myocardial infarction (AMI) [1, 2]. In such cases, achieving optimal reperfusion remains challenging because of the increased risk of thrombus burden, slow flow and suboptimal device delivery [3].

Guide catheter extension devices are indispensable for managing complex percutaneous coronary intervention (PCI), particularly in patients with tortuous coronary anatomy. Devices like the GuideLiner have demonstrated efficacy in navigating challenging vascular geometries. However, their performance may be limited in cases of severe tortuosity or ectasia. This report presents a unique case of ST‐segment elevation myocardial infarction (STEMI) in a patient with a severely ectatic and tortuous right coronary artery (RCA), where the use of a highly flexible guide catheter extension (GuidePlus, Nipro, Osaka, Japan) enabled successful recanalisation. This case underscores the importance of selecting appropriate devices tailored to complex coronary anatomies and highlights the utility of GuidePlus in such scenarios.

## 2. Case Presentation

**Figure 1:**
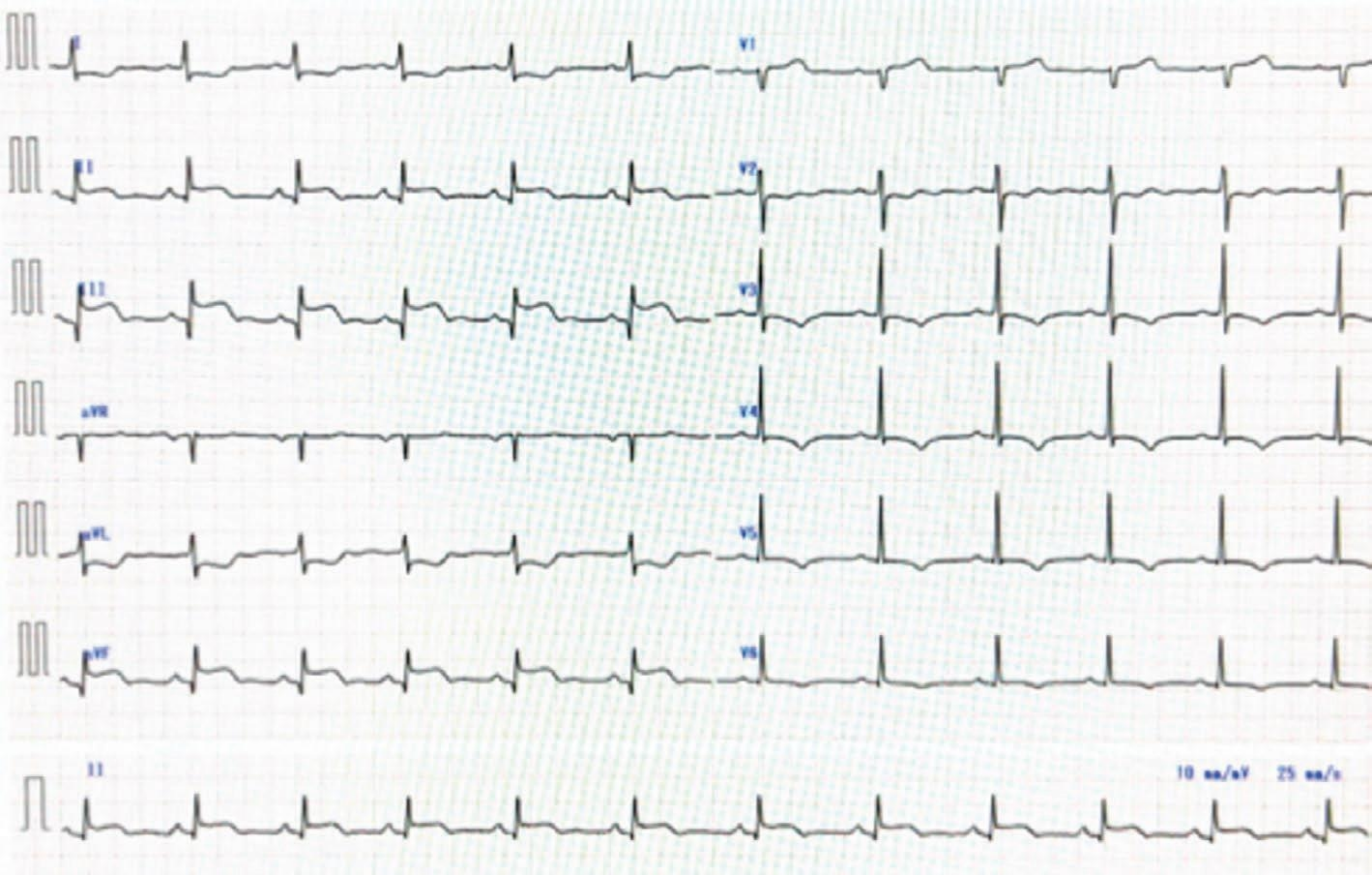
Admission electrocardiogram.

**Figure 2:**
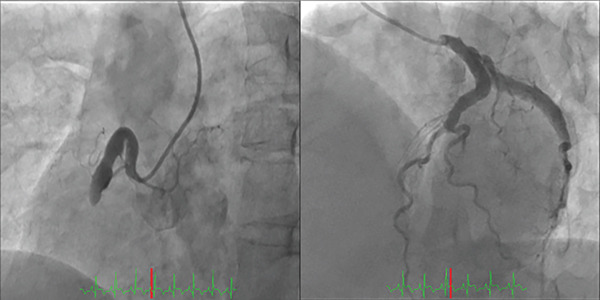
Preprocedural angiograms demonstrating a proximal occlusion of the right coronary artery with a ‘shepherd’s crook’ morphology. The left coronary artery exhibits proximal ectasia without significant stenosis.

**Figure 3:**
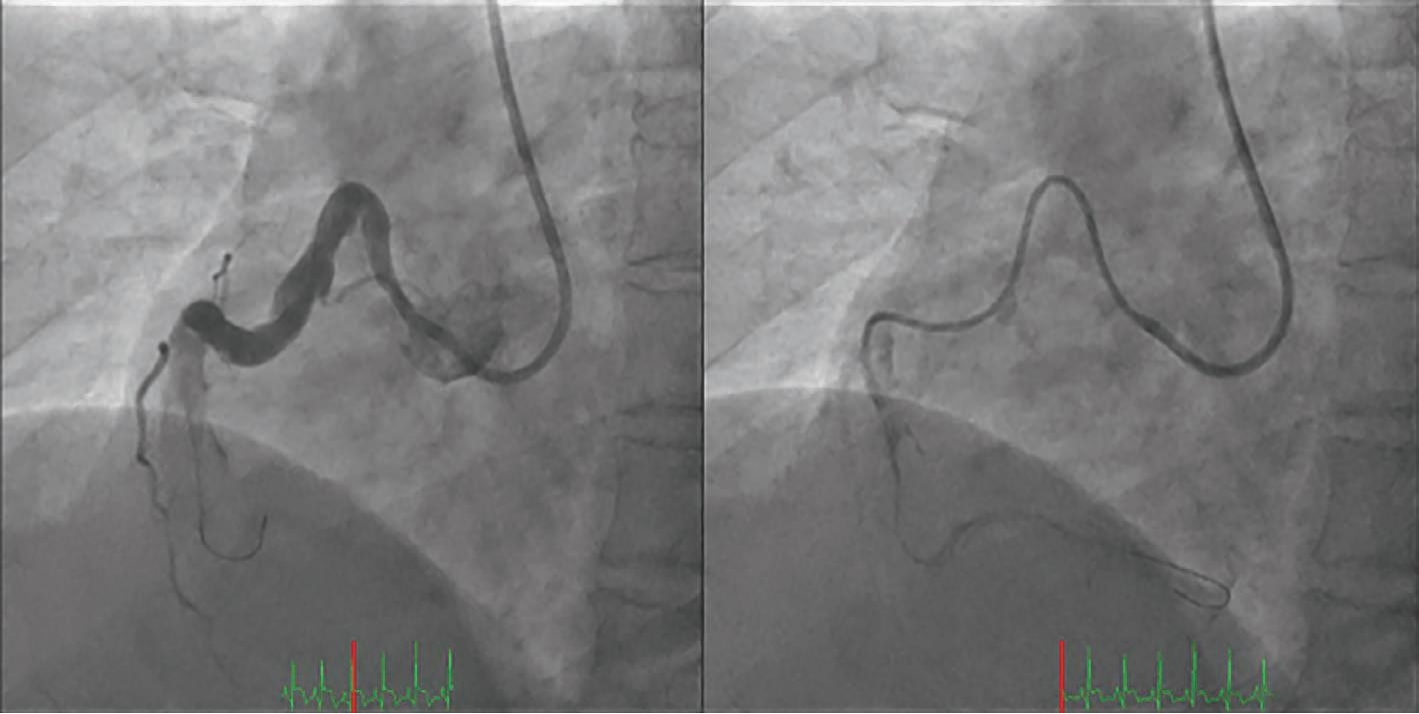
The right coronary artery was severely tortuous and ectatic and contained a massive thrombus. Manual aspiration thrombectomy was performed via the guiding catheter–GuidePlus system.

**Figure 4:**
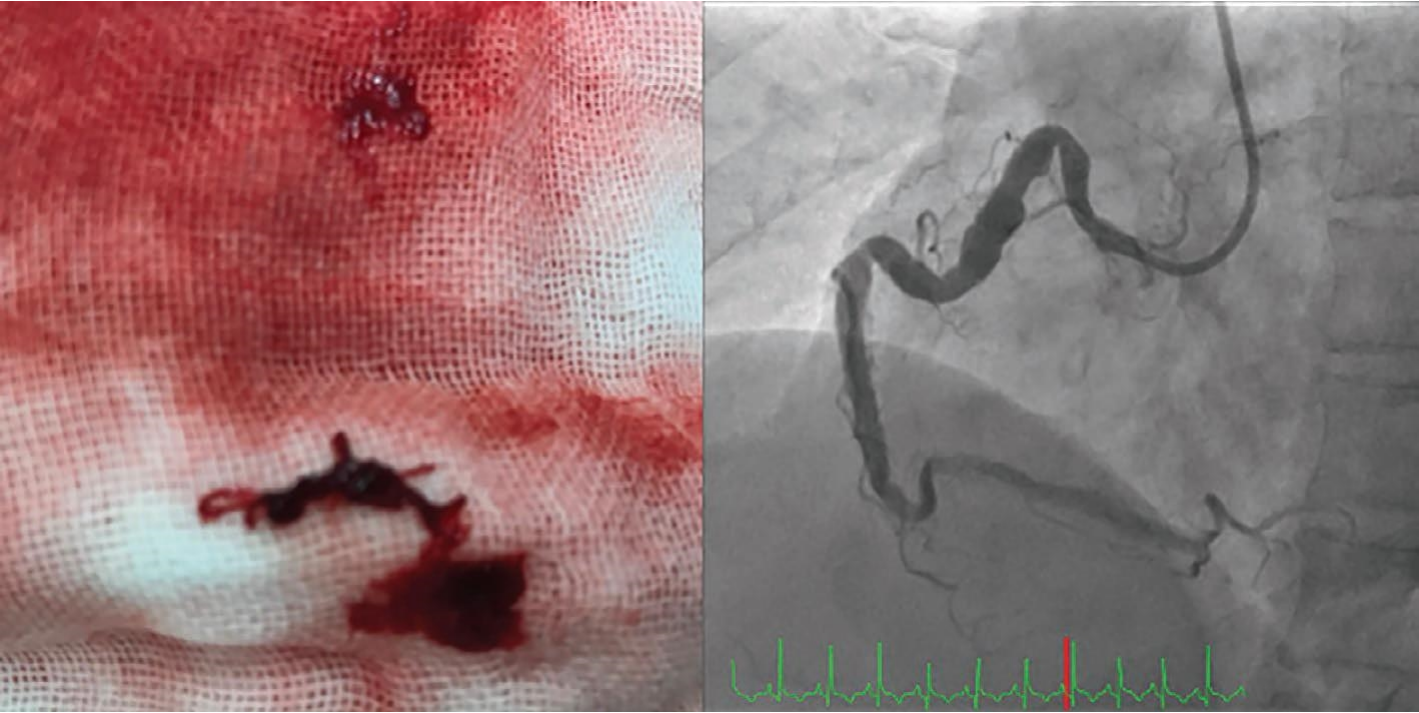
A large red thrombus was successfully aspirated, and the final angiogram demonstrated adequate recanalisation with only a small residual thrombus remaining.

A 45‐year‐old man presented with acute chest pain and was diagnosed with STEMI approximately 2 h after symptom onset. Electrocardiography performed on admission revealed ST‐segment elevations in Leads II, III and aVF (Figure 1). Coronary angiography revealed a complete thrombotic occlusion in the proximal segment of a severely ectatic (6.5 mm, 1.8× reference) and tortuous RCA, exhibiting a ‘shepherd’s crook’ morphology. The left coronary artery exhibited proximal ectasia without significant stenosis (Figure 2). Initial PCI was performed using a 6‐Fr Amplatz AL1.0 guide catheter (Hyperion AL1.0, Asahi Intecc Co., Ltd., Aichi, Japan) and a hydrophilic‐coated guidewire (Sion Black, Asahi Intecc). However, advancing the guidewire to the distal segment was impeded by severe tortuosity and friction within the ectatic artery. Attempts to deliver devices using a conventional guide catheter extension device (GuideLiner, Teleflex Inc., Wayne, Pennsylvania, United States) were unsuccessful, as the device failed to navigate the pronounced tortuosity of the proximal segment. To address these challenges, the GuidePlus guide catheter extension, a soft‐tipped, hydrophilic‐coated and highly flexible guide catheter extension, was introduced. Its exceptional crossability and flexibility enabled successful navigation through the tortuous vascular anatomy [4]. The GuidePlus guide catheter extension was advanced deeply into the RCA, positioning its distal tip as close as possible to the thrombus. Manual aspiration thrombectomy was then performed through the guiding catheter–GuidePlus system by connecting a 20‐mL syringe to the Y‐connector of the guiding catheter, allowing negative pressure to be transmitted to the distal tip of the extension catheter. Intracoronary urokinase (240,000 IU) was subsequently administered instead of a GP IIb/IIIa inhibitor (not routinely available in Japan) to dissolve the residual thrombus (Figure 3). Partial recanalisation was achieved after successful aspiration of a large red thrombus. Subsequently, a 4.0 × 28‐mm drug‐eluting stent (Ultimaster, Terumo) was deployed, and postdilatation was performed using an NC Trek 5.0 × 15‐mm noncompliant balloon at 12 atm to ensure optimal stent expansion. The final angiography confirmed satisfactory coronary flow with minimal residual thrombus (Figure 4). The patient recovered uneventfully and was discharged after 1 week. The patient remained symptom‐free during follow‐up with long‐term dual antiplatelet therapy comprising aspirin and prasugrel.

## 3. Discussion

Managing STEMI in patients with severely ectatic and tortuous coronary arteries poses significant technical challenges due to the increased risk of suboptimal device delivery, incomplete reperfusion and distal embolisation. The present case highlights how the GuidePlus catheter extension, with its exceptional flexibility and hydrophilic coating, can overcome these challenges and facilitate successful PCI outcomes.

CAE is a relatively rare condition that is frequently associated with a predisposition to thrombosis and AMI owing to sluggish flow and stasis [1, 2]. In such cases, conventional guide catheter extension devices often fail due to their limited flexibility and manoeuvrability, particularly in severely tortuous vascular anatomies. For instance, in our case, the GuideLiner device, which is widely used for PCI, was unable to navigate the extreme tortuosity of the proximal segment. This limitation underscores the need for alternative devices, such as GuidePlus, specifically designed for enhanced crossability and atraumatic navigation through complex anatomies [4]. The structural and material differences between the two guide catheter extension devices are summarised in Table 1.

**Table 1:** Comparison of GuidePlus and GuideLiner.

Feature	GuidePlus II EL (Nipro, Japan)	GuideLiner V3 (Teleflex, United States)
Tip design	Soft, tapered atraumatic tip	Rigid metal collar (improved transition)
Coating	Full‐length hydrophilic coating	Partial polymer liner
Extension cover	Smooth polymer sleeve with rounded transition	Metal collar with limited distal coverage
Material	Polyamide elastomer with stainless steel braid	Stainless steel coil with polymer liner
Available length (Japan)	35 cm	25, 35, 45 cm
Flexibility	High (soft polymer shaft)	Moderate (stainless‐steel reinforced)
Inner lumen (6 Fr)	0.056 ^″^ (1.42 mm)	0.056 ^″^ (1.42 mm)

The performance of GuidePlus in this case aligns with previous findings in which soft‐tipped, hydrophilic‐coated devices demonstrated superior efficacy in negotiating challenging vascular geometries [4]. In addition, prior case reports have suggested that guide catheter extension systems can facilitate aspiration in selected bailout scenarios when dedicated aspiration catheters cannot be delivered to the target lesion [5]. In the present case, the combination of severe ectasia and marked tortuosity (‘shepherd’s crook’ RCA) created substantial friction and limited device deliverability; therefore, the soft, hydrophilic GuidePlus provided not only crossability but also a practical route for manual aspiration close to the thrombus via the guiding catheter–extension system, followed by intracoronary thrombolysis. This approach enabled subsequent stent deployment and satisfactory final angiographic results with minimal residual thrombus. A summary of the clinical advantages and limitations of each device, including the GuidePlus, GuideLiner and Telescope, is presented in Table 2.

**Table 2:** Clinical comparison of guide extension devices.

Device	Major advantage	Limitation	Best use scenario
GuidePlus	Excellent flexibility and crossability, safe navigation	Slightly less backup support	Tortuous/ectatic RCA, complex PCI
GuideLiner	Strong support for device delivery	Poor flexibility	Straight or mildly tortuous vessels
Telescope (Medtronic)	Large inner lumen, long reach	Rigid tip	Large‐calibre proximal arteries

Although Kawasaki disease is a known cause of coronary aneurysms in adults, our patient had no childhood history of febrile illness, mucocutaneous symptoms or multiple aneurysms. The angiographic appearance was consistent with atherosclerotic ectasia rather than the sequelae of Kawasaki disease.

The combination of aspiration thrombectomy and intracoronary thrombolysis employed in this case also warrants discussion. Although the role of aspiration thrombectomy in STEMI has been debated because of conflicting trial results [6], it remains a valuable technique in selected cases involving a high thrombotic burden and ectatic arteries. Intracoronary urokinase administration further augmented thrombus resolution, a strategy supported by previous studies that demonstrated improved outcomes in similar settings [7, 8].

Currently, there is no consensus on the optimal management of markedly ectatic or aneurysmal coronary arteries. Surgical options, including aneurysm resection and coronary bypass grafting, have been described for the treatment of giant aneurysms. However, in this case, surgical intervention was considered less suitable given the single‐vessel involvement and acute STEMI presentation. Instead, catheter‐based strategies involving mechanical thrombectomy and intracoronary thrombolysis have been pursued.

In addition, aggressive antiplatelet therapy and, in some cases, anticoagulant therapy may be considered for secondary prevention owing to the ongoing risk of thrombus formation within the ectatic segments. Follow‐up coronary computed tomography angiography (CCTA) was planned to monitor potential thrombus recurrence and aneurysmal progression.

This report underscores the importance of tailored approaches in managing complex PCI cases. The successful use of GuidePlus highlights its potential as the preferred device in cases involving ectatic and tortuous coronary arteries. Future studies should focus on comparing guide catheter extension devices under various anatomical and clinical scenarios to optimise their use.

## Funding

No funding was received for this manuscript.

## Disclosure

Yasuyuki Toyama affirms that this manuscript is an honest, accurate and transparent account of this case. All authors approved the final version of the manuscript, and the corresponding author takes full responsibility for data integrity.

## Ethics Statement

The study complied with the ethical standards. IRB approval was not required for this case report.

## Consent

Written informed consent for publication was obtained from the patient for the purpose of this study.

## Conflicts of Interest

The authors declare no conflicts of interest.

## Data Availability

The anonymised data supporting this case report are available upon reasonable request from the corresponding author in compliance with ethical and privacy regulations.
